# Targeting Integrins in Cancer Nanomedicine: Applications in Cancer Diagnosis and Therapy

**DOI:** 10.3390/cancers11111783

**Published:** 2019-11-13

**Authors:** Ping-Hsiu Wu, Abayomi Emmanuel Opadele, Yasuhito Onodera, Jin-Min Nam

**Affiliations:** 1 Global Station for Quantum Medical Science and Engineering, Global Institution for Collaborative Research and Education (GI-CoRE), Hokkaido University, Sapporo 060-8638, Hokkaido, Japan; 2 Molecular and Cellular Dynamics Research, Graduate School of Biomedical Science and Engineering, Hokkaido University, Sapporo 060-8638, Hokkaido, Japan; abayomiopadele@gmail.com; 3 Department of Molecular Biology, Faculty of Medicine, Hokkaido University, Sapporo 060-8638, Hokkaido, Japan

**Keywords:** nanomedicine, nanoparticles, integrin, RGD peptide, active targeting, cancer diagnosis, drug delivery, radiotherapy, hyperthermia therapy

## Abstract

Due to advancements in nanotechnology, the application of nanosized materials (nanomaterials) in cancer diagnostics and therapeutics has become a leading area in cancer research. The decoration of nanomaterial surfaces with biological ligands is a major strategy for directing the actions of nanomaterials specifically to cancer cells. These ligands can bind to specific receptors on the cell surface and enable nanomaterials to actively target cancer cells. Integrins are one of the cell surface receptors that regulate the communication between cells and their microenvironment. Several integrins are overexpressed in many types of cancer cells and the tumor microvasculature and function in the mediation of various cellular events. Therefore, the surface modification of nanomaterials with integrin-specific ligands not only increases their binding affinity to cancer cells but also enhances the cellular uptake of nanomaterials through the intracellular trafficking of integrins. Moreover, the integrin-specific ligands themselves interfere with cancer migration and invasion by interacting with integrins, and this finding provides a novel direction for new treatment approaches in cancer nanomedicine. This article reviews the integrin-specific ligands that have been used in cancer nanomedicine and provides an overview of the recent progress in cancer diagnostics and therapeutic strategies involving the use of integrin-targeted nanomaterials.

## 1. Introduction

### 1.1. Cancer Nanomedicine

An increasing number of nanotechnologies have been applied to the screening, diagnosis, and treatment of cancer in the field of cancer nanomedicine. Since the first nanomedical cancer drug Doxil (liposomal doxorubicin) received approval by the food and drug administration of America (FDA) in 1995 [1], the number of new applications in cancer nanomedicine has increased. Compared with conventional cancer interventions, nanomedicine, which involves the nanoscale application of highly specific medical interventions, has unique features. For example, nanomedicine offers the ability to specifically target and greatly enhance the detection of tumors [2,3]. In cancer treatment, nanomedicine not only improves the therapeutic indexes of traditional medications but also provides innovative concepts for new treatment approaches [4]. Those appealing advantages have incentivized more scientists to undertake research in cancer nanomedicine, and these studies have contributed to the development of promising treatments for overcoming cancer in the future.

### 1.2. Characterization of Nanoparticles

To produce nanomedical agents for cancer diagnosis or treatment, scientists first select the nanoparticle (NP) platform on the basis of the therapeutic approach. The main structure of NP platforms can be divided into organic and inorganic materials. Organic NPs, such as liposomes [1], polymeric NPs [5], dendrimers [6], viral NPs [7], and exosomes [8], are usually used for drug delivery or gene therapy, and inorganic NPs include carbon-based NPs [9], metal-based NPs [10], mesoporous silica [11] and quantum dots (QDs) [12]. Over the last few decades, these NPs have become increasingly advanced with new designs and applications, such as functionalization for achieving stimuli-responsive effects [13]. For example, Gao et al. used inorganic NPs to induce heat after exogenous stimulation to trigger the release of cytotoxic agents [14]. In addition, due to their unique physical properties, inorganic NPs, such as metal-based NPs, can be used in innovative approaches, including the enhancement of radiotherapy [15] and the induction of hyperthermia in cancer cells [16].

**Figure 1:**
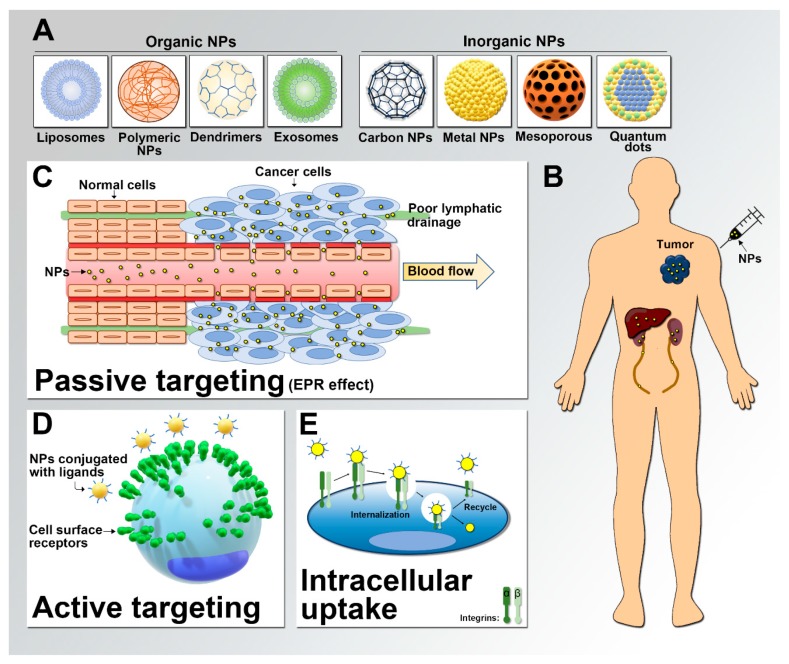
Uptake of nanoparticles (NPs). (**A**) Representative NPs used for cancer nanomedicine. (**B**) Accumulation of NPs in the human body. The organs that typically show the highest NP accumulation are the liver and kidney. (**C**) Schematic of passive targeting (enhanced permeability and retention (EPR) effects). NPs preferentially accumulate within tumors due to their leaky vasculature and poor lymphatic drainage. (**D**) Schematic of the active targeting of NPs conjugated with specific ligands that target surface receptors on cancer cells. (**E**) Schematic of intracellular uptake. This figure shows an example of the internalization of integrin-targeted NPs by cells through endocytosis after binding to integrins.

Subsequently, the delivery of NPs is considered, and there are two major approaches for transporting NPs to cancer cells: passive targeting and active targeting (Figure 1). By leveraging the pathophysiological processes in cancer (for example, leaky tumor vasculature, poor lymphatic drainage, and tumor microenvironment interactions), NPs can take advantage of the enhanced permeability and retention (EPR) effects to accumulate around tumoral tissue, and this process is called passive targeting [17]. The first-generation nanomedicine drugs, such as Doxil, Myocet (non- polyethylene glycosylated (PEGylated) liposomal doxorubicin) and DaunoXome (non-PEGylated liposomal daunorubicin), are EPR effect-based nanomedical drugs that have already been routinely used for treating patients [18]. However, the use of NPs through only passive targeting does not achieve the best therapeutic effects because the EPR effect applies not only to tumors but also to some normal tissues [19,20], such as hepatic or splenic tissue with fenestrated blood vessels, and leads to unexpected NP accumulation in these normal tissues. In addition, solid tumor tissues are heterogeneous neoplasms composed of different types of cells, including cancer cells, mesenchymal cells, endovascular cells, and immune cells [21]. This heterogeneity of tumoral tissue limits the ability of delivering NPs specifically to tumor cells.

To enhance the accumulation of NPs in cancer cells, scientists decorate NPs with targeting ligands that recognize specific receptors on the tumor cell surface, and this approach is called active targeting [22]. Active targeting effectively increases not only the tumor uptake of NPs independent of the EPR effect but also the ability of NPs to cross physiological barriers, such as the intestinal mucosa [23] or the blood–brain barrier [24]. Selecting the appropriate targeting ligand is critical for optimizing the efficiency of active targeting. Representative ligands used for the active targeting of NPs include antibodies, peptides, nucleic acids, sugars, and/or other small molecules [25]. In the past, antibodies have generally been selected as targeting moieties for use in nanomedicine due to their high specificity and wide availability [26]. However, the clinical use of antibody-based NPs is limited by certain features of the antibodies, such as the large size of antibodies, which impedes the effectiveness of surface conjugation [27], or the immunogenicity of antibodies, which leads to high clearance from the blood [28]. In addition, the easy degradation of antibodies during environmental changes (temperature, pH level, photostability, oxidation, etc.) is also a problem [29]. Peptides with smaller molecular sizes and simple three-dimensional structures do not have the disadvantages of antibody-based NPs. In addition, the synthesis of peptides is relatively simple and inexpensive compared with the production and cost of antibodies, which facilitates their translation to the clinic.

Research on nucleic acids is relatively more recent than that on peptides, and the lack of safety data and clinical reports on nucleic acids limits their application [30]. Sugars (such as saccharides, oligosaccharides, and polysaccharides) are larger than peptides, which affects their application for NP modification. Compared with other small-molecule agents, peptides are more specific to their targets because they are derived from linear protein sequences [31]. Therefore, peptides that can specifically bind to surface receptors on cancer cells, particularly integrin-targeted peptides, have attracted extensive attention.

### 1.3. Integrins in Cancer Nanomedicine

Integrins constitute a family of cell surface receptors that mainly facilitate cell-to-extracellular matrix (ECM) adhesion. Each integrin belonging to this family of heterodimeric transmembrane receptors is composed of an α subunit and a β subunit. Mammals have 18 α-subunits and eight β-subunits, and these subunits form 24 different integrins [32]. Various integrins play two major functions: attaching the cell body to the ECM and receiving signals transduced from the ECM. The extracellular domain of these integrins shows strong affinity for ECM proteins, including fibronectin, vitronectin, collagen, and laminin. After binding to ECM and clustering, these integrins also activate signal transduction pathways that mediate cellular signals related to cell growth, survival, division, and migration [33]. In contrast, the overexpression of certain integrins has been observed in highly malignant cancer cells (Table 1) and plays an important role in malignant properties, including cancer progression [34], invasion/metastasis [34], tumor angiogenesis [35], and even resistance to conventional cancer therapy [36,37]. These facts suggest that targeting integrins overexpressed in cancer cells is a feasible strategy for use in cancer nanomedicine.

The Arg-Gly-Asp (RGD) peptide is the most representative binding motif involved in the interactions of ECM proteins with integrins [73]. Since the RGD peptide was first discovered in 1984 [74], studies on integrin-targeted peptides in cancer diagnosis and treatment have become popular [75]. Interestingly, some of the artificial integrin-targeted peptide mimics act as antagonists that can inhibit integrin-mediated functions [76,77]. Through modification with integrin-targeted peptides, these NPs have been shown to exhibit not only high affinity to integrin-overexpressing cancer cells but also potential efficacy to suppress cancer progression through the inhibition of integrin-mediated functions [78,79].

In addition to the above-described features, the intercellular uptake and trafficking of integrins constitute another process that might be related to the efficacy of NPs. After binding to ECM ligands, integrins trigger ‘outside-in’ signals that promote downstream signaling to regulate the above-described cell behaviors [80]. Consequently, ligand-bound integrins are internalized by cells for focal adhesion turnover mainly by clathrin-mediated endocytosis and then transported to late endosomes or lysosomes [81]. In the acidic environment of late endosomes or lysosomes, some integrins are detached from the binding ligands, and the unbound free integrins are recycled back to the plasma membrane [80,82]. Incidentally, the required factors for regulating the recycling of integrins back to the plasma membrane are specifically upregulated in cancer cells and thus related to cancer progression [80,82]. Due to these characteristics (endocytosis and recycling of integrins), the integrin-targeted NPs are able to interact with integrins on the cell surface and are effectively internalized by the cancer cells together with the ligand [83], and some of these NPs accumulate in late endosomes and lysosomes [78].

## 2. Ligands Used in Integrin-Targeted NPs

By modification with integrin-targeted ligands on their surface, NPs can specifically target integrin-expressing cancer cells. As described above, the RGD motif is the first-discovered and the most widely studied integrin-targeted ligand [74] and can be recognized by integrins that are important for cancer progression and metastasis, including αvβ3-, αvβ5-, αvβ6-, αvβ8-, and α5β1-integrins [73]. In addition to the RGD motif, several non-RGD motifs have also been found to serve as specific integrin-target ligands and have characteristics that differ from those of RGD motifs (see below).

### 2.1. RGD-Based Integrin-Targeted Ligands

The RGD sequence has been found in many ECM proteins, including fibronectin [74], vitronectin [84], von Willebrand factor [85], osteopontin [86], and laminin [87]. The RGD-containing peptides can generally be divided into those with linear and those with cyclic structures. The cyclic RGD (cRGD) peptides display higher activity than the linear RGD peptides due to a less flexible conformational structure that resists proteolysis [88,89]. To enhance the biological properties and pharmacokinetics of RGD peptides, including their affinity, various strategies have been used to modify the structure of RGD peptides, such as altering their structure [90] and the stereochemical configuration of the constituent amino acids [91], introducing other amino acids to flank the RGD sequence [92], and *N*-methylation [93,94]. The modification of NPs with RGD peptides could increase their binding affinity to specific integrins. For example, Maltsev et al. transformed the long binding helix of an RGD ligand to an enzymatically stable cyclic peptide endowed with subnanomolar binding affinity toward the αvβ6-integrin receptor [90], and the resulting molecule could be used for intraoperative cytological assessment of bony resection margins in patients with head and neck cancer [95]. Cilengitide (Merck, Germany), an *N*-methylated cRGDfV derivative [c(RGDfNMeVal)], is a very potent antagonist of ανβ3-, ανβ5-, and α5β1-integrins [96]. Compared with other compounds, Cilengitide exhibits significantly higher binding affinity for these integrins [97]. Although Cilengitide failed to improve the treatment outcomes of glioblastoma multiforme in phase III clinical trials [98], NPs modified with Cilengitide show promising results in preclinical research [99]. Other well-known RGD peptides include cRGDfV [91] (the parent peptide of Cilengitide), cRGDfK [100], and RGD4C (ACDCRGDCFCG) [101]. In addition, iRGD (CRGDK/RGPD/EC), a relatively new compound, was produced to induce a multistep tumor-targeting process that differs from that of the other RGD peptides [102]. After binding to αv-integrins, iRGD is cleaved by a protease to expose the binding motif for neuropilin-1. Consequently, the iRGD-conjugated material is transferred from the integrins to neuropilin-1 and deeply penetrates into the tumor. This unique delivery method has been used in ongoing research in the field of cancer nanomedicine [103].

### 2.2. Non-RGD Integrin-Targeted Ligands

In addition to the RGD peptide, the Asp-Gly-Arg (NGR) peptide is an integrin-binding motif found in fibronectin [104]. On the basis of the structure of the NGR peptide, another peptide motif, isoDGR, which is found in fibronectin, was produced by in situ rearrangement to convert asparagine into iso-aspartate [105]. A survey of the binding affinity of integrin-targeted ligands to integrins revealed that the compound c(phgisoDGRk), which contains isoDGR, shows high affinity to ανβ6-, ανβ8-, and α5β1-integrins [97]. Several groups have used isoDGR ligands to modify NPs to target integrins [106,107]. Another non-RGD pentapeptide derived from the synergy domain of fibronectin is Ac-PHSCN-NH_2_, which was clinically developed under the trade name ATN-161 for the treatment of several solid tumors [36,108] due to its high affinity for α5β1-integrin and relatively lower affinity for αvβ3- and αvβ5-integrins [109]. Other integrin-targeted peptidomimetics, including SCH221153 (αvβ3- and αvβ5-integrin specific), BCH-15046 (αvβ3-, αvβ5-, and α5β1-integrin specific), SJ749 (α5β1-integrin specific), JSM6427 (α5β1-integrin specific), and A20FMDV2 (αvβ6-integrin specific), have been developed, and these have shown anticancer activities in preclinical models and when used on NPs [110,111].

In addition to the ECM-related peptides, tetraiodothyroacetic acid (tetrac), a thyroid hormone analog, has recently been used as an αvβ3-integrin-targeted ligand. Thyroid hormones induce tumor growth and angiogenesis via αvβ3-integrin [112,113]. On the basis of this concept, researchers have used tetrac to manufacture integrin-targeted NPs, which have recently shown promising results in preclinical studies [114,115].

## 3. Applications of NPs in Cancer Diagnosis

NPs have a wide range of applications, particularly in the field of molecular imaging. The introduction of molecular imaging to cancer diagnosis has provided a new approach for understanding tumor characteristics without depending on invasive diagnostic procedures [116]. Kircher et al. [117] defined molecular imaging as the noninvasive imaging of cellular and subcellular events. The advent of nanotechnology has led to the use of NPs in cancer diagnosis, and this targeted molecular imaging method offers a better approach for detecting cancer cells.

NPs with conjugated integrin-targeted ligands can be used to obtain images of integrins [118,119,120,121], which are overexpressed in many cancer cells and angiogenic vessels, as previously described. Among the advantages of imaging integrins, the following are specific to clinical cancer diagnosis: (i) the imaged integrins can be used to identify integrin-overexpressing tumors, which represent highly invasive or high-grade tumor disease for which a precise personalized cancer treatment can be applied; (ii) tumor imaging can lead to the early detection of metastatic disease; and (iii) the imaging of tumors can reveal tumor neoangiogenic activity that requires antiangiogenic therapies. The composition of the integrin-targeted NPs is an important factor for obtaining precise and useful integrin images. In addition, selecting the appropriate diagnostic tool for imaging integrins is another important issue to consider. Several imaging modalities have been employed for accurate cancer diagnoses, but different studies have shown that positron emission tomography (PET) imaging [2], magnetic resonance imaging (MRI) [122], fluorescence reflectance imaging (FRI) [123], and fluorescence molecular tomography (FMT) [3] are particularly effective methods for imaging integrin-targeted NPs due to their high spatial resolution and ability to capture images in real time.

In this section, we review the representative means for fabricating and characterizing integrin-targeted NPs for use in cancer diagnosis and then describe the associated techniques.

### 3.1. Fabrication of Integrin-Targeted NPs for Cancer Diagnosis

To obtain highly precise images of integrins targeted by NPs, several factors should be considered. Montet et al. [123] used cRGD-conjugated fluorescence-based NPs to detect integrin-expressing cancer cells in tumor-bearing mice through MRI, FRI, and FMT images. These researchers suggested that (i) the expression level of integrins in tumor cells, (ii) the pharmacokinetics of NPs (which should have a sufficient half-life in blood to slowly escape from the vasculature over a long circulation time), and (iii) the vascularized nature of tumors (through which NPs enter the cancer cells efficiently) are the factors that influence RGD-conjugated NPs to allow the efficient imaging of integrins.

In addition to the detection of integrin-expressing cancer cells at their site of origin, NPs can be used for the early detection of metastatic cancer cells, which makes NPs attractive for use in cancer diagnosis. A significant proportion of deaths result from cancer metastases [124]. Several studies have suggested that early metastatic cancer cells can be targeted with NPs [125,126], although the targeting of metastatic cancers with nano-objects, which have a relatively small size and low vascularization, has proven to be a Herculean task. To achieve this goal, Peiris et al. fabricated chain-shaped NPs with c(RGDfC) conjugated on their surfaces for tumoral vascular targeting [3]. Nanochain technology was deployed to fabricate four iron oxide nanospheres [127] that were then fused with cRGD to create chain-shaped NPs, and the resulting NPs were linearly assembled via chemical reaction [128]. Due to the high metastatic potential of 4T1 breast cancer cell lines [129], Peiris et al. used 4T1 tumor-bearing mice to evaluate the uptake of the NPs by metastatic cancer cells [3]. These researchers reported that the cRGD-conjugated chain-shaped NPs have the potential to detect metastatic tumors in addition to primary tumors.

Similarly, another study fabricated RGD peptides with green fluorescent zinc oxide nanowires (ZnO-NWs) [130]. Zinc oxide is a biocompatible multifunctional material with excellent piezoelectric and pyroelectric properties that can be used as a medical fluorescent material [131,132]. In addition, the low toxicity and biodegradable properties of nanomaterials in the human body are also important features of ZnO and make it a great candidate for use in cancer nanomedicine [133]. To produce compact and useful ZnO-based NPs for cancer diagnosis, Hong et al. synthesized specific green fluorescent ZnO-NWs and further conjugated ZnO-NWs with the c(RGDyK) peptide to target αvβ3-integrin [130]. These researchers demonstrated the usefulness of ZnO-NWs for the cancer-targeted optical imaging of U87MG human glioblastoma cells with high αvβ3-integrin expression. However, αvβ3-integrin-negative cells, such as MCF-7 human breast cancer cells, did not show fluorescence signals.

Biocompatible QDs conjugated with RGD peptides have also been considered nanomaterials for detecting the tumor vasculature [134]. The introduction of QDs for biological and optical imaging was largely based on the ability to deliver these nanosized objects to cancer cells [134,135]. In fact, Cai Weibo et al. reported the use of QDs conjugated with the RGD peptide (QD705) for imaging the αvβ3-integrin-positive tumor vasculature in living mice [134]. The study also reported that during angiogenesis, the overexpression of αvβ3-integrin was detected in the tumor vasculature, and this observation paved the way for using QDs for optical integrin-targeted imaging in cancer diagnosis. Similarly, Smith et al. investigated the tumor neovasculature in mice by conjugating the QDs with RGD peptide, which bind to luminal endothelial cells, to capture images of αvβ3-integrin [136]. These researchers revealed that QDs bind aggregately rather than individually, which indicates the unexpected distribution pattern of αvβ3-integrin in the tumor neovasculature. However, the challenge of using QDs is that their large size causes them to be retained in the vascular system, which could restrict their exudation from the blood vessels and lead to diffusion to cells distant from the vessels. It has been reported that these effects could potentially induce toxicity to normal tissues and thus reduce the imaging efficiency and sensitivity of QDs [137]. To correct this anomaly, an ultra-small sized luminescent silver sulfide (Ag_2_S) NP was developed, and the resulting Ag_2_S QDs induced negligible toxicity in tissues [138]. In contrast, Lin et al. synthesized ultrasmall superparamagnetic iron oxide NPs (USIO-NPs) using a coprecipitation method and conjugated the USIO-NPs with RGD peptides to target integrins [139]. Their study further revealed that RGD-conjugated USIO-NPs have the potential to differentiate human cancer tumors and cells with different integrin expression levels, such as MCF-7, A549, HT-29, and HT-1080 cells [139].

### 3.2. Techniques for Imaging Integrin-Targeted NPs

As described above, PET [2], MRI [122], FRI [123], and FMT [3], among others, have proven to be effective methods for imaging integrin-targeted NPs. With the aid of a small-animal PET system, tumor imaging has been performed with iodine-124 dimeric cyclic RGDyk upconversion nanophosphors [^124^I-(cRGDyk)_2_-UCNPs] in a U87MG tumor model [2]. Images of αvβ3-integrin were taken after the administration of a conjugated radionuclide during PET scanning, which lasted for approximately 1 h.

In contrast, MRI technology is considered an effective imaging modality due to its high spatial resolution and its non-reliance on ionizing radiation (IR). In fact, Vargas et al. suggested that diffusion-weighted MRI can be used to detect aggressiveness in certain malignancies [140]. Goswami et al. conjugated cRGD peptides to a vertex-differentiated contrast agent, [closo-B12]^2−^ (CA-12), and investigated its ability to target αvβ3-integrin following in vivo MRI analysis [122]. The study confirmed the ability of cRGD-conjugated CA-12 to selectively target αvβ3-integrin while inducing negligible toxicity in the site injected with the contrast agent. This kind of study might improve MRI by overcoming its low specificity in several situations (such as the false-positive rate of 10% for breast cancer [141]) and are thus likely to have a significant impact on cancer diagnosis in the future.

Additionally, optical imaging is considered a suitable preference for imaging cellular and molecular processes due to its high sensitivity. In further prospective research, Akers et al. [142] reported that for imaging αvβ3-integrins, RGD-conjugated NPs can be used to delineate tumor-induced angiogenesis by optical imaging. They suggested that when developing new molecular agents for cancer imaging, the animal model selected might influence the outcome. The use of multiple animal models is important for assessing the robustness of these molecular agents with high tumor-to-surrounding tissue contrast. 

## 4. Applications in Cancer Treatment

### 4.1. Drug Delivery

A conventional method of treating cancer involves chemotherapy. However, systemic toxicity, severe side effects, and the inability to achieve sufficient drug accumulation in the tumor remain the current challenges associated with the use of chemotherapeutic agents [143,144]. To overcome these challenges, Tian et al. suggested that innocuous, tissue-specific, and noninflammatory (i.e., avoiding the induction of side effects caused by innate immune activation) delivery technologies should be developed and translated into clinical practice for cancer therapy [145]. Further studies revealed that with the aid of nanotechnology, specifically designed NPs, which act as delivery vehicles, can control therapeutic processes in cancer therapy [146,147]. In fact, several studies on the use of ligand-mediated “smart drug delivery systems” have revealed that therapeutic agents can be more specifically transported to tumor sites by targeting αvβ3-integrins on cancer cells, and these types of agents have proven to be efficient in a cancer therapy regime [148,149,150,151]. Several typical chemotherapeutic agents, such as doxorubicin [145,152,153,154], cisplatin [149], and paclitaxel [155,156,157], have been combined with RGD-conjugated NPs to target integrin-overexpressing tumors.

Doxorubicin is an important cancer therapeutic agent that is currently used as the standard treatment for many neoplastic diseases. However, it has also been reported that the dose-dependent cardiac toxicity of this agent, which leads to cardiomyopathy, has limited its clinical use [158]. To overcome the short biological half-life and adverse effects of doxorubicin, polymer-based NPs conjugated with RGD peptides were developed to deliver doxorubicin directly to a tumor site [159,160,161,162]. Moreover, a similar study using RGD-conjugated polymer-based NPs further modified these NPs with organelle-targeting ligands [163]. In the study, the NPs that targeted the nucleus, which were achieved with RGD4C-PEO-b-P(CL-Hyd-DOX) (RGD ligand poly(ethylene oxide)-block-poly(ε-caprolactone) with doxorubicin conjugated to the core using pH-sensitive hydrazone bonds), induced the highest cytotoxic response in doxorubicin-sensitive cancer cells, and the mitochondrion-targeted NPs, which were obtained with RGD4C-PEO-b-P(CL-Ami-DOX) (RGD ligand poly(ethylene oxide)-block-poly(ε-caprolactone) with doxorubicin conjugated to the core using stable amide bonds), induced the highest cytotoxic response in doxorubicin-resistant cancer cells [163]. In addition to polymer-based NPs, Tian et al. manufactured iRGD-conjugated exosomes (iRGD-Exos) by engineering immature murine dendritic cells with the aim of producing drug-carrier NPs that induce low inflammatory and toxicity [145]. The iRGD-Exos were loaded with doxorubicin by electroporation to create iRGD-Exos-Dox. Compared with doxorubicin alone or Exos-Dox without iRGD conjugation, iRGD-Exos-Dox exerted a superior cytotoxic effect in αv-integrin-positive breast cancer cells in both in vitro and in vivo experiments. The study suggests a possible clinical approach for using an integrin-targeted exosome-based drug delivery system for the treatment of tumor disease [145].

In addition to doxorubicin, cisplatin is a widely used anticancer drug [164]. It has been estimated that only a small proportion (only 1% or less) of the Pt(II) compound cisplatin is delivered to the cells and binds to DNA, whereas a significant proportion (approximately 90%) is targeted toward proteins and low-molecular weight biomolecules [165,166]. To overcome this disparity, a novel method was developed to convert Pt(IV) complexes into prodrugs that can be intracellularly activated by reduction to generate Pt(II), and as a result, a large fraction of platinum can be delivered to the cancerous cells [166]. Graf et al. [149] synthesized a polymeric NP system that consists of an encapsulated Pt(IV) prodrug and cRGD peptides targeted to αvβ3-integrin on cancer cells and performed in vitro tests. Their results revealed that the synthesized NPs exhibited enhanced cytotoxicity compared with cisplatin administered at its conventional dosage in prostate and breast cancer cell lines.

Other chemotherapeutic drugs, such as paclitaxel, which has the trade names Taxol and Abraxane [155,156]; cetuximab, which is also known as Erbitux [167]; and temozolomide, which is an oral alkylating chemotherapeutic drug [168], have shown strategic promise in cancer therapy when loaded onto integrin-targeted NPs, which has resulted in an improved treatment efficacy.

Different therapeutic agents induce cytotoxic effects in cancer cells via different mechanisms and thereby produce drug resistance in different ways [169]. Hence, the simultaneous combination of different chemotherapeutic agents for the treatment of tumor disease has been used to improve the therapeutic outcomes. However, the therapeutic outcomes of combination chemotherapy remain unsatisfactory due to discrepancies in tumor uptake and their different pharmacokinetic profiles [170]. These obstacles can be overcome by nanomedicine. Several studies used integrin-targeted NPs and loaded them with two different therapeutic agents. For example, some researchers loaded topotecan (TPT) and quercetin (QT) on mesoporous silica NPs for the treatment of integrin-expressing breast cancer cells [171], another group loaded paclitaxel and cisplatin onto RGD-conjugated lipid-polymer NPs for the treatment of lung tumor [172], and another study combined doxorubicin with c-Myc small interfering RNA (siRNA) and loaded these onto RGD-conjugated NPs [173]. These new-generation NP-based drugs provide a promising future for improving chemotherapy.

### 4.2. Radiotherapy, Hyperthermia Therapy, and Photodynamic Therapy

In addition to serving as drug delivery carriers, NPs have other applications, such as enhancers or producers of therapeutic effects by themselves. These types of NPs are mainly metal-based NPs, which can cause greater damage directly in cancer cells following external excitation. In this section, we discuss the application of integrin-targeted NPs in radiotherapy, photothermal therapy (PTT), magnetic hyperthermia therapy (MHT), and photodynamic therapy (PDT).

#### 4.2.1. Radiotherapy

Radiotherapy is one of the standard and effective cancer therapies based on IR. Over the last decades, clinical and in vitro studies have revealed that elements with a high atomic number (Z) can enhance the external radiation effect [174,175]. The application of IR to high-Z material generates several types of emissions, including scattered X-rays/photons, photoelectrons, Compton electrons, Auger electrons, and fluorescence photons, which can enhance the radiation effect in the area around the high-Z material [176]. With recent advances in nanomedicine, the application of high-Z metals as radiosensitizers has attracted the interest of researchers in radiation oncology. Among the high-Z metals, gold (Au, Z = 79) is the element most often used as an NP platform. Gold NPs (AuNPs) possess several advantages over other materials: (i) good biocompatibility, as indicated by the ease with which AuNPs enter the human body without inducing harmful effects [177]; (ii) the straightforward nature of the synthesis of different-sized AuNPs [178]; and (iii) the easy functionalization of AuNPs by conjugating ligands to its surface [179]. These characteristics have inspired individuals to conjugate integrin-targeted ligands to the surface of AuNPs, which illustrates the concept of “targeted radiosensitizers”. We have reported the radiosensitizing effects of RGD-conjugated polyethylene-glycosylated AuNPs (RGD/P-AuNPs) on integrin-overexpressing breast cancer cells [78]. Our study showed that RGD/P-AuNPs are efficaciously internalized into integrin-overexpressing cancer cells subjected to an increase in radiation-induced DNA damage. Interestingly, the IR-induced invasiveness [180] was also suppressed by the RGD/P-AuNPs [78]. Several studies have revealed that some cancer cells that survive radiotherapy might exhibit enhanced invasiveness (IR-enhanced invasiveness) [181,182] or acquire an invasive phenotype [183], which might lead to a higher proportion of distal recurrences after radiotherapy. Because integrins play important roles in cancer invasion and migration [184], it is not surprising that integrin-targeted NPs may influence the IR-induced invasiveness of cancer cells. However, although invasion is an important hallmark of cancer cells [185], most studies on nanomedicine have not focused on the effect of cancer invasion after integrin-targeted NP treatment. Hence, the evaluation of cancer cell invasiveness in studies on integrin-targeted NPs would provide important and useful information for researchers in the field of cancer biology and nanomedicine. Consistent with our in vitro study, an in vivo study performed by Liang et al. [186] demonstrated the capacity of RGD-conjugated AuNPs to increase the therapeutic effect of IR. These researchers produced c(RGDyC)-AuNPs and reported that c(RGDyC)-AuNPs are more highly accumulated in tumors compared with non-RGD-conjugated AuNPs. Their results also showed that the use of c(RGDyC)-AuNPs followed by radiotherapy effectively reduces the tumor size. In addition to conventional X-ray irradiation, Enferadia et al. [187] used protons (particle radiotherapy) combined with c(RGDfK)-conjugated ultrasmall AuNPs (1.8-nm diameter) in a murine glioma cell model and compared the results with those obtained with kilovolt and megavolt X-ray therapy. Their results showed that the c(RGDfK)-AuNPs enhanced the efficacy of all the combined treatments, but no obvious differences were found between the different radiation modalities.

In addition to sensitizing cells to external IR treatment, another approach is the conjugation of radiolabeled peptides to AuNPs. Vilchis-Juárez et al. produced c[RGDfK(C)] conjugated-^177^Lu-Labeled AuNPs (^177^Lu-AuNP-RGD) and validated their therapeutic effect in glioma-bearing mice [188]. Their results showed that Lu-AuNP-RGD delivered the most highly absorbed tumor radiation dose in tumor cells compared to that of Lu-AuNPs or Lu-RGD. The uptake of Lu-AuNP-RGD by nontargeted organs was low in the treated mice. As described above, the therapeutic efficacy of both external radiotherapy and radioactive treatment can be enhanced by integrin-targeted AuNPs.

#### 4.2.2. Hyperthermia Therapy

Hyperthermia therapy (also known as thermotherapy) generally described the use of heat (i.e., a temperature higher than the normal body temperature (>37 °C)) to treat disease [189]. The use of hyperthermia as a method for treating cancer has a long history, dating back to approximately 3000 B.C. in ancient Egypt [190]. The temperature used in hyperthermia cancer treatments can be categorized as nonlethal (39 to 42 °C) or lethal (>42 °C). At a nonlethal temperature, tumor oxygenation is improved, which makes cancer cells more sensitive to radiotherapy or chemotherapy [191]. At lethal temperatures, cancer cells are more greatly damaged than normal cells because heat cannot be readily dissipated by the circulating blood in tumor tissue [192]. Although hyperthermia research continues to improve adjuvant or direct therapy, the means of heating tumor tissue to an effective temperature remains a critical problem. Conventional external heat sources, such as microwave or ultrasound, have limited by their inability to conduct heat to a high depth in tumor tissues [193]. With the advancement of nanomedicine, several metal NPs were found to have a high capacity to induce heat through energy transduction [194]. This phenomenon introduced the “inside-out” hyperthermia therapy because the heat source, an NP, is placed in the cancer cells. According to their different trigger approaches, these types of therapy can be generally identified as PTT and MHT. Photothermal therapy usually utilizes external near-infrared (NIR) radiation (wavelength from 750 to 2500 nm) to irradiate the light-absorbing NPs that accumulate in cancer cells. The absorption and scattering of NIR radiation in the human body are minimal but can increase the temperature of light-absorbing NPs to higher than 42 °C [195]. The most characterized light-absorbing NP platform is based on AuNPs due to their incomparable absorbance of NIR radiation [196]. Several studies have shown promising results regarding the use of cRGD-conjugated AuNPs in PTT for the treatment of breast cancer cells [197], melanoma cells [198], and human glioma cell-bearing mice [199]. In addition to AuNPs, NPs of copper sulfide (CuS) also show good photothermal properties and can thus be used in PTT. A study used cRGD-conjugated CuS NPs to treat human gastric tumor cell-bearing mice by PTT, and the results showed that cRGD-CuS NPs selectively entered primary and lymph node metastatic tumor cells to treat cancer without obvious side effects [200]. In addition to using the hyperthermia effect of Cu to kill cancer cells, novel Cu-based NPs were recently developed to induce cancer cell cytotoxicity using another approach [14]. Vinyl azide, a cytotoxicity agent, is encapsulated into c(RGDfE)-conjugated hollow copper sulfide NPs. Upon NIR irradiation, the local temperature increases to trigger the vinyl azide to rapidly release N_2_ bubbles, and these N_2_ bubbles instantly explode to destroy the neovasculature that expresses αvβ3-integrin and further induce necrosis of the surrounding tumor cells. This notion, which was inspired by PTT, provides a novel approach in cancer nanomedicine for the future development of more effective therapies.

In contrast to PTT, MHT utilizes an alternating magnetic field (AMF) instead of irradiating light to generate heat in magnetic NPs. Iron oxide NPs (or ferrite NPs) are the most studied magnetic NPs to date. A study showed that cRGD-conjugated iron oxide NPs can be used for tumor detection by MRI and induce MHT to treat cancer cells [201]. However, although MHT is the oldest and best-known external localized heat therapy, the use of AMF is complicated, and the heating efficacy of MHT is unclear compared with that of PTT, the application of MHT faces challenges. A study used RGD peptide-conjugated magnetosomes (synthesized by magnetotactic bacteria, which show efficacy for MHT) to treat human prostatic and uterine cancer cells by PHT and MHT excitation, respectively [202], and their results showed that PHT was much more efficient than MHT in both cellular and in vivo models. Therefore, identification of the appropriate approaches for inducing hyperthermia therapy should be carefully considered.

Although the use of hyperthermia as the single cancer treatment modality still faces many challenges [192], recent studies have yielded promising results from the combination of hyperthermia with other treatment modalities, such as chemotherapy [203], radiotherapy [204], and immunotherapy [205]. With the assistance of integrin-targeted NPs, the application of hyperthermia to increase the therapeutic efficacy of other treatments might become more feasible and can possibly improve cancer therapy.

#### 4.2.3. Photodynamic Therapy

Photodynamic therapy (PDT) for cancer deploys the use of photosensitizing agents that are injected into the bloodstream and transmitted to cancerous cells and thereby expose a tumor to a wavelength of light that causes the direct killing of cancer cells or shrinkage of the tumor volume [206,207]. To specifically deliver photosensitizing agents into cancer cells, several studies have used integrin-targeting NPs as carriers. Wang et al. conjugated carboxyl functionalized iron oxide NPs with a fibronectin-mimetic peptide (PR_b, [KSSPHSRN(SG)_5_RGDSP]) [208], which contains RGD and another fibronectin sequence, Pro-His-Ser-Arg-Asn (PHSRN) [209], that binds integrins. The study revealed that the combination of a second-generation photosensitizing agent, Pc 4, with these RGD-conjugated iron oxide NPs showed promising advantages compared with ordinary Pc 4 in the treatment of head and neck cancer. In addition, the combination of Pc 4 with these NPs also improved the MRI contrast [208]. Li et al. used c[RGDfK(Ac-SCH_2_CO)] peptides conjugated with albumin-based NPs with a photosensitizer IRDye 700DX to treat ovarian cancer in three dimensional (3D) culture, and the results showed a significant cytotoxic effect in cancer cells [210]. Other studies have also shown promising results regarding the use of integrin-targeted NPs to deliver photosensitizing agents to cancer cells [211,212,213].

## 5. Issues of Integrin-Targeted NPs

The modification of NPs with integrin-targeted ligands has become a useful practice in cancer nanomedicine, but several issues remain to be considered. For example, integrin-targeted NPs might have a lower efficacy for the treatment of low-integrin-expressing cancer cells. To overcome this problem, some groups have attempted to mix different ligands to enhance the targeting ability of the NPs; for example, some researchers have conjugated RGD and folate (to target the folate receptor, which is overexpressed in many tumor cells) together [214] or have used an anti-vascular endothelial growth factor (VEGF) aptamer together with RGD on NPs [215]. Another problem is the localization of AuNPs in cells. As described above, integrin-targeted NPs mainly accumulate in late endosomes and lysosomes [78]. This localization suggests that NPs appear to work in lysosomes, but in some situations, the function of the lysosomes might decrease NP efficacy. For example, some studies have used NPs to deliver siRNA into cancer cells for gene therapy, but the siRNA might be digested in lysosomes [216]. To overcome this problem, several strategies for facilitating endosome escape, such as ion pair formation, the “proton sponge effect”, destabilization of the endosome membrane, and the hydrophobic modification of the NPs, have been researched [217]. These types of efforts might compensate for the shortage of integrin-targeted peptides and achieve the goal of using NPs to reach the anticipated goals.

## 6. Conclusions

The use of integrin-targeted NPs significantly improves the efficacy of cancer nanomedicine. The benefit of these integrin-targeted NPs has been comprehensively examined in various applications of cancer nanomedicine. We believe that the use of integrin-targeted NPs will be widely used in the future in clinical settings, where they will improve the efficacy of cancer diagnosis and treatment.

## Figures and Tables

**Table 1:** Arg-Gly-Asp (RGD) peptide-binding integrins in cancer cells.

Integrin	Binding Ligands	Specific Functions in Cancers	Associated Cancers (Detected in Clinical Studies)
α5β1	Fibronectin	Increases tumor progression [38] Increases cancer invasion [39] Mediates resistance to radiotherapy [36]	Head and neck cancer [40,41]
	Vitronectin		Non-small cell lung cancer [42]
	Fibrinogen		Breast cancer [43]
	Osteopontin		Prostate cancer [44]
			Ovarian cancer [45]
			Melanoma [46]
ανβ3	Fibronectin	Increases tumor progression [47] Increases lymph node metastasis [48] Increases bone metastasis [49] Is involved in cancer immune evasion [50]	Glioma [51]
	Vitronectin		Head and neck cancer [40]
	Fibrinogen		Non-small cell lung cancer [52]
	Osteopontin		Lung cancer brain metastases [53]
	Tenascin		
	Thyroid hormone T4		Gastric cancer [54]
			Pancreatic cancer [48]
			Prostate cancer [55]
			Melanoma [46]
αvβ5	Fibronectin	Increases tumor progression [56] Is involved in glioma invasion [57]	Lung cancer brain metastases [53]
	Vitronectin		Non-small cell lung cancer [52]
	Fibrinogen		Gastric cancer [54]
	Osteopontin		Prostate cancer [55]
αvβ6	Fibronectin	Promotes hepatic tumorigenesis [58] Increases tumor progression [59] Increases lymph node metastasis [60] Mediates resistance to chemotherapy [61]	Head and neck cancer [62]
	Vitronectin		Non-small cell lung cancer [63]
	Fibrinogen		Breast cancer [64]
	Osteopontin		Lung cancer brain metastases [62]
	Tenascin		Gastric cancer [65]
			Pancreatic cancer [55]
			Colon cancer [66]
			Endometrial cancer [67]
			Ovarian cancer [68]
			Basal cell carcinoma [69]
αvβ8	Fibronectin	Is involved in cancer immune evasion [70] Mediates resistance to chemo- and radiotherapy [71]	Head and neck cancer [72]
	Vitronectin		Non-small cell lung cancer [52]
	Fibrinogen		Prostate cancer [55]
	Osteopontin		

## List of Abbreviations

**AuNPs**	**Gold NPs**
3D	three dimensional
CA-12	[closo-B12]^2−^
cRGD	Cyclic RGD
ECM	Extracellular matrix
EPR	Enhanced permeability and retention
Exos	Exosome
FDA	The food and drug administration of America
FMT	Fluorescence molecular tomography
FRI	Fluorescence reflectance imaging
IR	Ionizing radiation
Lu-AuNP-RGD	RGD conjugated-^177^Lu-Labeled AuNPs
MHT	Magnetic hyperthermia therapy
MRI	Magnetic resonance imaging
NIR	Near-infrared
NP	Nanoparticle
PDT	Photodynamic therapy
PET	Positron emission tomography
PEGylated	polyethylene glycosylated
PHSRN	Pro-His-Ser-Arg-Asn
PTT	Photothermal therapy
QDs	Quantum dots
QT	Quercetin
RGD	Arg-Gly-Asp
RGD/P-AuNPs	RGD-conjugated polyethylene-glycosylated AuNPs
siRNA	Small interfering RNA
Tetrac	Tetraiodothyroacetic acid
TPT	Topotecan
USIO-NPs	Ultrasmall superparamagnetic iron oxide NPs
VEGF	Vascular endothelial growth factor
Z	Atomic number
ZnO-NWs	Zinc oxide nanowires
